# Fast and simple voltammetric sensing of avanafil in the pharmaceutical formulation by using unmodified boron-doped diamond electrode

**DOI:** 10.5599/admet.2357

**Published:** 2024-06-27

**Authors:** Hoshyar Saadi Ali, Hemn A. H. Barzani, Yavuz Yardım

**Affiliations:** 1 Department of Analytical Chemistry, Faculty of Science, Van Yuzuncu Yil University, 65080 Van, Turkey; 2 Department of Medical Laboratory Science, College of Health Science, Lebanese French University, Erbil, Iraq; 3 Department of Analytical Chemistry, Faculty of Pharmacy, Van Yuzuncu Yil University, 65080 Van, Turkey

**Keywords:** Electrochemical sensing, drug analysis, square-wave voltammetry

## Abstract

**Background and purpose:**

Erectile dysfunction is a common issue among adult males involving difficulty in maintaining an erection, and it is often treated with fast-acting, low-side-effect drugs like avanafil (AVN), among other phosphodiesterase-5 inhibitors. Hence, developing fast, simple, and sensitive methods to detect AVN is crucial.

**Experimental approach:**

This study conducts an electroanalytical inquiry and provides a new voltammetric method for accurately analyzing AVN utilizing a boron-doped diamond (BDD) electrode without any modifications.

**Key results:**

In the Britton-Robinson buffer (BR, 0.04 mol L^-1^, pH 4.0), cyclic voltammetry showed a clearly defined and irreversible anodic peak at around +1.44 V relative to Ag/AgCl. The pH of the solution was shown to have an impact on the voltammetric signals of the oxidation peaks. A good linear response for AVN quantification was achieved using square-wave voltammetry. This was done in a 0.04 mol L-1 BR (pH 4.0) solution at a potential of +1.33 V (*vs.* Ag/AgCl). The method exhibited a wide dynamic range of 0.5 to 30.0 μg mL^-1^ (1.0 to 62 μmol L^-1^) and a low limit of detection of 0.14 μg mL^-1^ (0.29 μmol L^-1^). The method proposed demonstrated suitability for determining AVN content in pharmaceutical formulations. The accuracy of the approach was demonstrated by comparing the results obtained using the developed method with those achieved using the UV-Vis spectrometry method.

**Conclusion:**

Our method simplifies the analytical process by eliminating the need for electrode modification, reducing both time and resource requirements while enhancing overall feasibility.

## Introduction

**Scheme 1.**
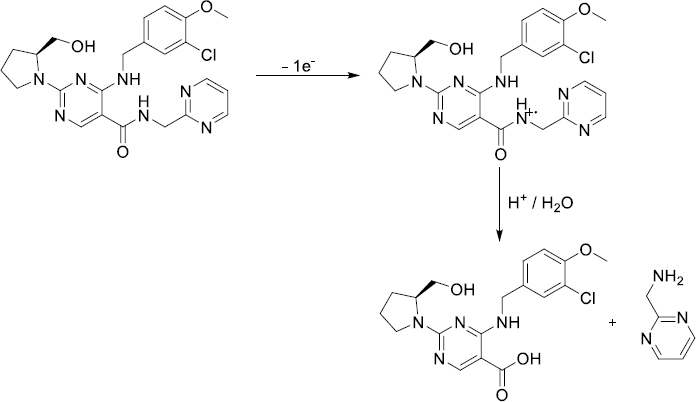
Proposed oxidation pathway of AVN on the BDD electrode surface.

Erectile dysfunction (ED) is a prevalent sexual disorder in adult males characterized by chronic or recurring difficulty in achieving and/or sustaining a penile erection adequate for sexual activity [1]. Moderate to severe ED affects an estimated 5-20 % of men throughout their sexual lives. There are a number of phosphordiesterase-5 (PDE5) inhibitors that are employed to treat ED, including vardenafil, tadalafil, sildenafil, and avanafil (AVN). AVN is particularly popular because it operates quickly and has few visible adverse effects compared with the other inhibitors of PDE5 [2]. AVN, (S)-4-((3-chloro-4-methoxybenzyl) amino)-2-(2-(hydroxymethyl) pyrrolidin-1-yl)-N-(pyrimidin-2-ylmethyl) pyrimidine-5- carboxamide is shown in Scheme 1 [3]. Specifically, AVN blocks the action of the PDE5 enzyme. The European Medicines Agency (EMA) (2013) and the United States Food and Drug Administration (FDA) (2012) both gave their approval for the treatment of ED. The therapy of ED has made extensive use of AVN. Ejaculatory latency is enhanced because it promotes blockage of the serotonin transporter, such that the postsynaptic cleft becomes more active with serotonin. AVN is distinguished by its quicker initiation of impacts and its reduced pictorial adverse effects compared to other PDE5 inhibitors. AVN exhibits limited solubility in water, leading to its reduced bioavailability of 38 to 41 % [4]. The typical approach to enhance the oral bioavailability of weakly water-soluble medications is by formulating them as nanoparticles [5]. These nanoparticles, as well-defined by Junghanns and Müller (2008), are subdivisions with a size smaller than 1 μm [6].

During the literature review conducted for AVN, it was found that there are only a few analytical techniques available for its evaluation. These methods can be categorized into three primary groups: HPLC, utilized for analyzing AVN in blood samples [7] and pharmaceutical formulations [8-13]; UV spectrophotometry for AVN analysis [14], as well as LC-MS/MS estimation of AVN in human plasma [15-19], LC-quadrupole-TOF-MS/MS [20], and electrochemistry [21-23].

Extensive studies conducted in the past twenty years have revealed that boron-doped diamond (BDD) electrodes, a recently developed carbon-based material, have garnered significant consideration in various fields, including biological and material science, chemical analysis, environmental investigations, among others [24-31]. BDD exhibits remarkable characteristics without undergoing any chemical alteration, in comparison to both noble metals (such as Au and Pt) and common sp2 carbon electrode materials (like pyrolytic graphite, glassy carbon, carbon paste, and others). The BDD electrode offers numerous advantages, including an extended electrochemical potential range in both non-aqueous and aqueous environments, consistent corrosion resistance, low background current, minimal pollutant adsorption, and remarkable mechanical durability [24,28,32,33].

Based on our comprehensive review of the literature, only three research papers in the field of electrochemistry have been used for the estimation of AVN. Notably, all of these investigations employed modified electrodes. Our study is the first to estimate AVN using bare BDD electrodes without any modification. The proposed method's applicability for determining AVN in pharmaceutical formulations was successfully demonstrated. Furthermore, the outcomes achieved for pharmaceutical formulation will undergo comparison via those derived from the UV-spectrophotometric approach application.

## Experimental

### Reagents

No further processing was performed on the AVN (ReagentPlus®, 98.0 % standard reference) purchased from ChemScene LLC (USA) before its use in this experiment. The samples were prepared utilizing analytical-grade reagents and water was filtered using a Millipore Milli-Q system. The electrolytes used were Britton-Robinson (BR) buffer, phosphate buffer solution (PBS), HNO_3_ (0.1 mol L^-1^), and H_2_SO_4_ (0.1 mol L^-1^). The pH range of the buffers was 2 to 12, and the reagents were analytical-grade (obtained from Sigma-Aldrich, Germany). The AVN was dissolved in a 0.1 mol L^-1^ HCI solution to create a stock solution with a concentration of 1.0 mg mL^-1^. While not in use, this item was stored in a refrigerator at 4 °C and protected from sunlight in the lab. A diluting of the stock solutions in the provided supporting electrolyte allowed for the measurement of the AVN concentrations.

### Instrumentation and measurements

The electrochemical evaluation was carried out on an Autolab Type III apparatus (Metrohm Autolab B.V., Utrecht, The Netherlands), and the data was collected and analysed with the assistance of the GPES software (version 4.9). In order to correct the baseline of all the stripping voltammograms, the moving averages algorithm was employed. Before this, a Savicky and Golay algorithm was utilized to smoothen the voltammograms, with a peak width of 0.01 V. The three-electrode setup employed in the electrochemical cell used BDD as a working electrode (Windsor Scientific Ltd.) with a diameter of 3 mm and a boron doping level of 1000 ppm, an Ag/AgCl (3 mol L^-1^ NaCl) reference electrode (BAS, Model RE-1) and a Pt counter electrode (BAS, MW-4130, USA). At 25 °C, the pH readings were taken by a WTW inoLab720 pH meter (Xylem Analytics, Germany) that used a combination glass electrode.

Implementing an activation method similar to the ones outlined in the literature [34] is necessary. As per our earlier research work, the BDD surface underwent pretreatment with it [35-37]. Each day of the experiment commenced with subjecting the BDD electrode to anodic (APT) and cathodic (CPT) pretreatment in 0.5 mol L^-1^ H_2_SO_4_. This was done using a dedicated voltammetric cell with an applied voltage of +1.8 and -1.8 V. In the time between each measurement, the equipment was hand-cleaned. In our previous study, we found that the selected activation protocol enhanced the surface activity of the BDD electrode. The electroactive surface area was determined to be 0.036 cm^2^ [38]. After gently rubbing the electrode for less than a minute with a moist, smooth cleaning cloth (BAS polishing pad), the residual byproducts were cleaned away using deionized water. This protocol ensured the electrode surface is free of AVN and its oxidation byproducts.

The electrochemical oxidation performance of AVN, as well as the reaction kinetics occurring at the BDD electrode within the supporting electrolyte, were initially investigated by cyclic voltammetry (CV). Utilizing the square-wave voltammetry (SWV) mode, we then optimized the experimental parameters, including the supporting electrolyte and SWV settings, while simultaneously enhancing the sensitivity and selectivity of the AVN analysis. Additionally, the technique's analytical performance and practical applicability were evaluated using the same pulse approach, *i.e.*, SWV. Before doing any quantifiable measurement of AVN by SWV, the three electrodes were immersed in electrochemical cell containing AVN and BR buffer (pH 4.0). Subsequently, anodic scanning was conducted using the SWV technique, spanning from 0 to 1.60 V. The quantitative measurement of AVN was achieved by optimised parameters of 12 mV scan increment, 60 mV pulse amplitude, and 50 Hz frequency using the SWV method.

In order to obtain comparison outcomes, a quantitative analysis of AVN in tablets was performed utilizing a UV/Vis spectrophotometric technique. The experiment utilized an AE-S90 UV-Vis spectrophotometer (AE Lab Guangzhou Co., Ltd, China) equipped with a quartz cell of 1 cm in diameter. The recording of absorption spectra was conducted using 0.1 mol L^-1^ HCl as the blank solution, covering a wavelength range of 200 to 800 nm. The regression equation was generated utilizing a calibration graph that was produced using the absorbance value of AVN at its maximum absorption wavelength of 245 nm as well as its corresponding concentrations in μg mL^-1^.

### Preparation of sample

Avanair® was procured online and utilized as the sample for AVN analysis in the form of a fixed-dose combination tablet. Every tablet contained 100 milligrams of AVN. The method involved using a mortar and pestle to finely grind ten pills at a precise weight. A Variable quantity of the acquired powder, corresponding to 5.0 mg AVN, was weighed and placed within a 50 mL calibrated dark flask with 0.1 mol L^-1^ HCl. Complete dissolution was achieved by continually stirring for around 5 min. A volume of 100 μL of the solution (comprising 0.04 mol L^-1^ BR buffer at pH 4.0) was introduced into the voltammetric cell using a micropipette. The analysis was performed after the addition of AVN at concentrations of 1, 2.5, 5, 10, 15, and 20 μg mL^-1^. The AVN content throughout the sample was determined using the standard addition technique.

The next phase demonstrated the proposed method's applicability to human urine samples. Urine samples were collected from a healthy 33-year-old male volunteer who had not taken any drugs. A 9.0 mL urine sample was placed in a test tube and spiked with 1.0 mL of an AVN standard working solution (1.0 mg mL^-1^). After thorough mixing for 1 min, 0.05 mL of the mixture was transferred to a voltammetric cell and diluted to 10 mL with the chosen supporting electrolyte (0.04 mol L^-1^ BR at pH 4.0). The urine sample without AVN served as a blank. AVN detection was then performed using the standard addition method.

## Results and discussion

**Figure 1.**
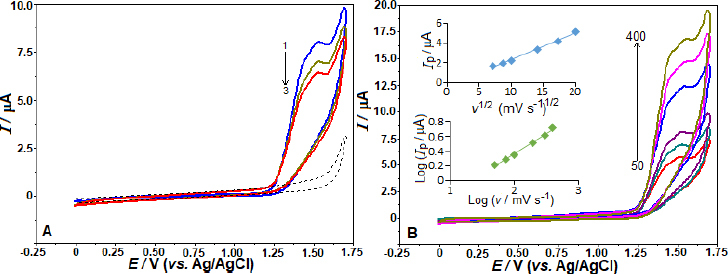
The BDD electrode was used to evaluate a concentration of 50 μg mL^-1^ of AVN in BR buffer with a pH of 4.0. Cyclic voltammograms were performed at a scan rate of 100 mV s^-1^ (A), and the scan rate was adjusted in subsequent experiments to 50, 75, 100, 200, 300, and 400 mV s^-1^ (B). The recorded scans are shown in sequence by the arrow. We may see the background current as dotted lines. B: The inset displayed the linear dependences of *i*_p_ with respect to *v*^1/2^ and log *i*_p_ with respect to log *v*.

The electrochemical oxidation of AVN on the BDD electrode surface was explored by the CV. Three successive CV cycles were taken for 50 μg mL^-1^ AVN in a 0.04 mol L^-1^ BR buffer (pH 4.0) at a potential range from 0 to 1.70 V. The scan rate was 100 mV s^-1^. On the first anodic scan, AVN exhibited a clear anodic oxidation peak at approximately 1.44 V, as shown in Figure 1A. However, since the reverse scan did not show a cathodic signal for AVN in the same potential range, it can be concluded that AVN's redox activity is irreversible in these experiments. The current peak height decreases with cycling, which can be explained by the passivation of the electrode and the surface contamination resulting from the AVN oxidation.

The kinetics of AVN oxidation at the BDD electrode were determined by employing the CV technique in a 0.04 mol L^-1^ BR buffer at a pH of 4.0. The scanning rates ranged from 50 to 400 mV s^-1^, with a total of six scanning rates. This experiment aimed to investigate the effect of potential scan rates (*v*) on the anodic peak current to 50 μg mL^-1^ AVN, and the obtained cyclic voltammograms are shown in Fig 1B. With an increase in the scan rate, anodic peak current height is increased.

The linear correlation among the square root of the scan rate (*v* ^1/2^) and the oxidation peak current (*i*_p_) of the AVN was determined using the equation *i*_p_ / μA = 0.276 *v*^1/2^ (mV s^-1^)^1/2^ - 0.431, via a correlation coefficient (*r*) of 0.994. The following equation, with a correlation coefficient of 0.996, was utilized to establish the linear relationship among log *i*_p_ and log *v*: log *i*_p_ / μA = 0.553 log *v* / mV s^−1^ - 0.739. The observed slope, approximately 0.553, falls within the expected range of 1.0 to 0.5 for methods influenced by adsorption and diffusion effects. To evaluate the number of electrons (*n*) involved in AVN oxidation on the BDD electrode, the equation *αn* = 47.7/(*E*_p_-*E*_p_/2) was used, with *E*_p_ (electrochemical potential difference) determined to be 77 mV. From this equation, the number of electrons involved in the reaction can be calculated. With the assumption of charge transfer coefficient (α) being 0.5, n equals 1.24, which approximates 1. This finding aligns with the observations reported in a prior investigation about the oxidation mechanism of AVN [22].

**Figure 2.**
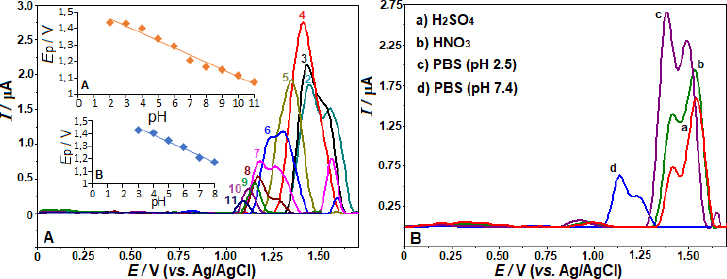
SW voltammograms were used to examine a 5 μg mL^-1^ AVN solution in BR buffer (pH 2.0-11.0) (A) and different electrolytes at different pH levels (B) at the BDD electrode. SWV parameters: frequency, 50 Hz; step potential, 8 mV; pulse amplitude, 30 mV. The inset in A contains two graphs that depict the correlation between the linear relationships of *E*_p_ / V and pH within the ranges of 2-11 (A) and 3.0-8.0 (B).

By employing the SWV, we examined the impact of various electrolytes and pH on the AVN oxidation peak current at the BDD electrode. The effects were investigated in BR buffer solution with a pH range of 2.0-12.0 and 5 μg mL^-1^ AVN concentration. The observed trend in Figure 2A indicates that the peak potentials shift towards less positive values as the solution pH increases from 2.0 to 11.0. From pH 2 to 4, the peak current increased; however, at pH 4, the peak current began to gradually decline. Under these experimental conditions, there was no oxidation peak at pH 12.0. The linear correlation between AVN anodic peak potentials and pH in the range of 3.0 to 8.0 can be described by the equation *E*_p_ / V = -0.055 pH + 1.609 (*r* = 0.991). The data suggests that an equivalent amount of electrons and protons is involved in the reaction mechanism, as seen by the slope of 0.055 V/pH being close to the theoretical value of 0.059 V. According to the suggested oxidation mechanism, a radical cation is produced when AVN is oxidized at the nitrogen atom of the amide group [22,23]. Scheme 1 depicts the oxidation mechanism of AVN.

As shown in Figure 2B, the SW voltammograms for different electrolytes are presented. The anodic peak potentials of +1.56, 1.57, 1.39, and 1.15 V were obtained in 0.1 mol L^-1^ HNO_3_, H_2_SO_4_, PBS pH 2.5, and 7.4, respectively. The corresponding peak currents were 1.92, 1.54, 2.61, and 0.63 μA, consistent with the findings reported in BR buffer. Also, from Figures 2A and 2B, a peak splitting and/or additional current peaks appearing as shoulders are evident in working pHs other than pH 4 and 5. The voltammogram on the BDD electrode with 5 μg mL^-1^ AVN in BR buffer at pH 4.0 produced the most impressive results, showing the highest peak current and a well-defined, sharp peak (Figure 2 A and B). Consequently, this voltammogram was chosen for further examination.

**Figure 3.**
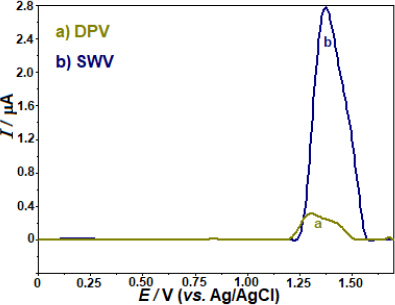
In the BDD electrode, DPV (a) and SWV measurements were taken at a concentration of 5 5 μg mL^-1^ AVN in BR buffer (pH 4.0). DPV parameters: modulation amplitude; 50 mV; step potential; 8 mV and modulation time; 0.05 s. SWV parameters: frequency 50 Hz; step potential 8 mV and pulse amplitude 30 mV.

The sensitivity of two pulse techniques, SWV and differential pulse voltammetry (DPV), in identifying the anodic peak currents of AVN was evaluated. When comparing the anodic peak currents of AVN measured by SWV and DPV, the experimental results showed that the former was around 8.6 times higher (Figure 3). Following this, more investigation will be done using the SWV method.

SWV parameters, including frequency (*f*), pulse amplitude (Δ*E*_sw_), and step potential (Δ*E*_s_), were optimized to achieve the highest peak current and enhance sensitivity. All other factors were kept constant except one. Via the Δ*E*_s_ and Δ*E*_sw_ held at 8 mV and 30 mV, respectively, the *f* value varied in a 25 to 150 Hz range. The anodic peak current increased as the *f* value increased. On the other hand, the peak width was significantly widened for *f* > 50 Hz. By applying this finding to SW voltammetric responses, we have shown that it gives higher analytical selectivity. The remaining experiments used *f* = 50 Hz. In the 30 to 70 mV region, the impact of the ΔEsw (remaining parameters: ΔEs= 8 mV, f = 50 Hz) on the intensity of the oxidation peak current was also measured. The oxidation peak current increased linearly as a function of Δ*E*_sw_ in the experimental range. A noticeable widening of the SW voltammograms was observed when Δ*E*_sw_ was raised to values greater than 60 mV. While the Δ*E*_s_ varied from 6 to 16 mV, and the other parameters remained constant (*f* = 50 Hz, Δ*E*_sw_ = 60 mV), the voltammetric signal that was recorded rose unless it reached a value of 12 mV, and then it increased at a slower rate from 12 to 16 mV. On the other hand, the SW curves exhibited a broadening when the Δ*E*_s_ values were more than 12 mV. That is why Δ*E*_s_ = 12 mV was selected for further experiments. Hence, the optimal SWV parameters for AVN oxidation on the BDD electrode in a 0.04 mol L^-1^ BR buffer at pH 4.0 were obtained with *f* = 50 Hz, Δ*E*_s_ = 12 mV, and Δ*E*_sw_ = 60 mV. The aforementioned graphic does not portray these values.

**Figure 4.**
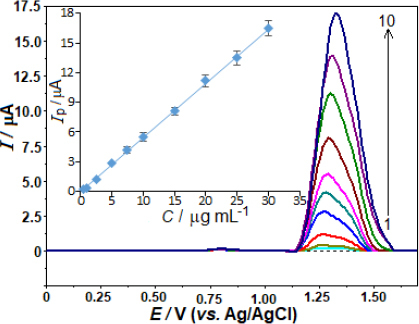
SW voltammograms for AVN levels of 0.5, 1.0, 2.5, 5.0, 7.5, 10.0, 15.0, 20.0, 25.0 and 30.0 μg mL^-1^ (1-10) in 0.04 mol L^-1^ BR buffer at pH 4.0 on the BDD electrode. SWV parameters are as follows: frequency, 50 Hz; step potential, 12 mV; pulse amplitude, 60 mV. Inset shows the corresponding calibration plot for the quantitation of AVN.

The evaluation of the analytical performance involved a relationship between the oxidation peak current and the AVN concentrations. The evaluation was conducted after optimising the working conditions, encompassing the experimental settings and instrumental variables. In accordance with this, the AVN stock solution was added to the voltammetric cell in small amounts at regular intervals, and the SWV responses were recorded after each addition. Concentration ranging from 0.5 to 30.0 μg mL^-1^ (1.0 to 62 μmol L^-1^) was employed to obtain the SW curves. The calibration curve for this purpose is depicted in Figure 4. A strong linear correlation is observed between the SW voltammograms, which exhibit an AVN peak potential of +1.33 V, following the successive addition of AVN: *i*_p_ / μA = 0.550 *C* / μg mL^-1^ - 0.058 (*r* = 0.999, *n* = 10).

The variables *i*_p_, *C*, *r*, and *n* represent the anodic peak current, AVN concentration, correlation coefficient, and number of experiments, respectively.

The analytical curve determined the limit of detection (LOD) at 0.14 μg mL^-1^ (0.29 μmol L^-1^) as well as the limit of quantification (LOQ) at 0.46 μg mL^-1^ (0.95 μmol L^-1^). A 3 s/m approach was used to evaluate the LOD. The slope (*m*) of the corresponding analytical curve and the standard deviation (s) of the 10 least concentration observations within the calibration range were taken into account.

To the best of our information, previous studies have not utilized bare working electrodes for the determination of AVN. Our analysis, employing non-modified BDD electrodes, yielded results with greater sensitivity in terms of the LOD (0.29 μmol L^-1^) compared to previous outcomes [21]. However, other studies show greater sensitivity than our study [22,23] (Table 1). Nonetheless, the proposed methodology demonstrates superior practicality, cost-effectiveness, and efficiency in measuring AVN compared to earlier research efforts.

**Table 1.** Comparison among the developed work and the previously reported studies.

Analyte	Electrode	Linear range, μmol L^-1^	LOD, μmol L-^1^	Reference
AVN	ZnO-NPs/MWCNT/CP	2.7–0.33	0.71	[21]
AVN and nimodipine	NiO-NPs/poly(sulf)/PGE	0.05 – 1.0	0.016	[22]
AVN and doxorubicin	TiO_2_/MWCNTs/CPE	0.01 – 8.0	0.003	[23]
AVN	BDDE	1.0-62	0.29	This study

ZnO-NPs/MWCNT/CP; Zinc oxide nanoparticles multi-walled carbon nanotubes carbon paste electrode, NiO-NPs/poly(sulf)/PGE; NiO nanostructured/ sulfanilamide polymeric film pencil graphite electrode, TiO_2_/MWCNTs/CPE; titanium dioxide nanoparticles multi-walled carbon nanotubes carbon paste electrode, BDDE; Boron-doped diamond electrode

The precision of the developed approach was evaluated by quantifying its consistency under identical circumstances, both within and across different days. Ten separate measurements of 0.5 μg mL^-1^ AVN solution were used to evaluate the repeatability of the anodic peak current's amplitude within a single day. The results were reproducible with an RSD of 5.6 %. After that, the RSD was calculated by measuring the peak current response over five consecutive business days. The results showed an inter-day RSD of 7.2 %. These results prove that a BDD electrode is suitable for reproducible determination of AVN in pharmaceutical formulations and samples.

**Figure 5.**
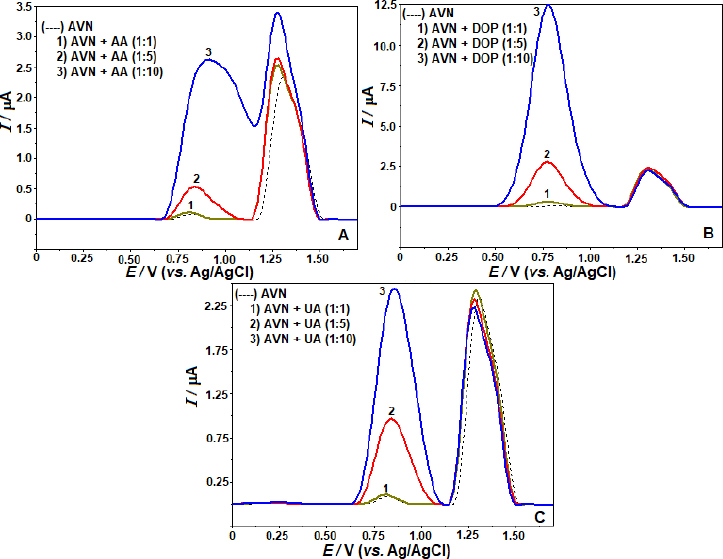
SW voltammograms of AVN (5.0 μg mL^-1^) mixture in the existence of (A) equimolar concentration, 5 and 10-fold excess AA, (B) equimolar concentration, 5 and 10-fold excess DOP and (C) equimolar concentration, 5 and 10-fold excess UA. Other operating conditions as indicated in Figure 4.

Preliminary tests were conducted using SWV at a concentration of 5 μg mL^-1^ AVN under identical experimental conditions as the real samples to detect possible interfering compounds. Pharmaceutical formulations or biological components are the primary sources of their presence. The acceptance limit was determined at a concentration that caused an inaccuracy of approximately ±5% in the oxidation peak current of the AVN. Sodium (Na^+^), potassium (K^+^), calcium (Ca^2+^), magnesium (Mg^2+^), zinc (Zn^2+^), iron (Fe^3+^), and titanium (Ti^4+^) ions, among others, were found to have no discernible impact on the oxidation peak current of AVN while applied in 10-fold excess. The AVN oxidation peaks were also unaffected by sugars such as maltose, glucose, sucrose, and fructose, even in a concentration 10× higher than the concentration of AVN. It was also found that pharmaceutical formulation components such microcrystalline magnesium stearate, cellulose, and cornstarch had little impact on the oxidation current responses of AVN. The effects of ascorbic acid (AA), dopamine (DOP), and uric acid (UA), potentially present in body fluids, were investigated at molar concentrations of 1:1, 1:5, and 1:10 (AVN solution:interfering compound). At UA, DOP, and AA concentrations relevant to this study, we find that individual solutions do not influence the anodic peak currents of AVN (Figure 5). According to the findings, the created approach exhibits high selectivity and opens up possibilities for using it on actual samples.

**Figure 6.**
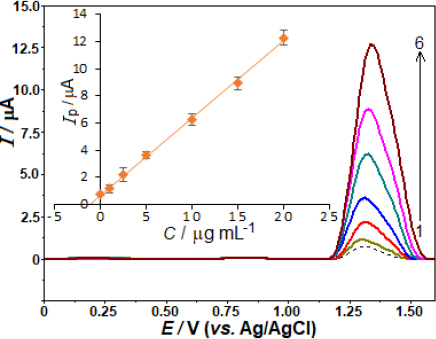
SW voltammograms were obtained for the diluted drug sample (shown by dashed lines) and afterward addition standard amounts of the drug to accomplish final concentrations of 1.0, 2.5, 5.0, 10.0, 15.0, and 20.0 μg mL^-1^ AVN in a 0.04 mol L^-1^ BR buffer at pH 4.0. on the BDD electrode. Inset depicts the result of analysis by standard addition method. Other operating conditions as indicated in Figure 4.

By measuring AVN in pharmaceutical formulations already on the market, we were able to ascertain the method's potential usefulness. Methods for conducting experiments, such as collecting samples and taking measurements, are detailed in the Experimental Section. We examined the tablet samples without extracting, filtering, or evaporating any materials. As shown in Figure 6, the sample and the standards that were used for analysis are shown in the SW voltammograms. Additionally, the graphical assessment is presented, which includes the equation [*i*_p_ / μA = 0.595 *C* /μg mL^− 1^ + 0.614 (*r* = 0.998)]. The amount of AVN in the pill was found to be 103.2 mg (RSD of 3.8 %), which is quite similar to the manufacturer-reported figure (100.0 mg) after accounting for the sample's consecutive dilutions.

A voltammetric cell was used to perform spike/recovery studies with commercial samples to confirm that the newly discovered approach could be used in real-world applications. Voltammetric responses were evaluated after adding 10 mL of sample solution to standard AVN solutions with concluding amounts of 1, 5, and 10 μg mL^-1^, as shown in Table 2. According to Table 2, there was no noticeable impact of the matrix on the proposed method.

**Table 2.** Pharmaceutical formulation samples spiked with AVN standard solutions, and their recovery values measured using the suggested voltammetric method

Concentration of AVN, μg mL^-1^			Recovery ± RSD, %
Added^a^	Expected^a^	Found^a, b^	
0	−	1.03	- ± 3.8
1.0	2.03	1.95	96.1 ± 3.5
5.0	6.03	6.19	102.7 ± 3.1
10.0	11.03	10.82	98.1 ± 2.9

^a^Concentration in the measured solution

^b^Average of three replicate measurements

**Figure 7.**
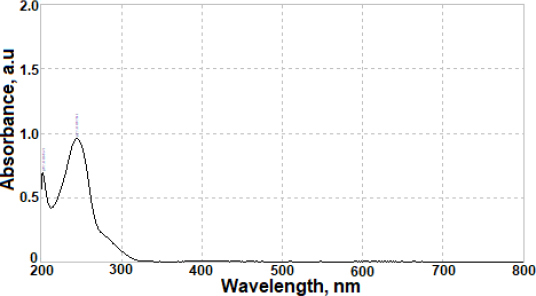
Absorption spectrum for 10 μg mL^-1^ AVN in 0.1 mol L^-1^ HCl using 1 cm quartz cell

The results of the developed voltammetric procedure for commercial pharmaceutical formulations were juxtaposed with those successfully acquired using a straightforward UV spectrophotometry method. A typical absorption spectrum of AVN in 0.1 mol L^-1^ HCl is shown in Figure 7. Calibration studies were conducted over the AVN concentration range of 1.0 to 20.0 μg mL^-1^ (equivalent to 2.1 to 41 μmol L^-1^), with measurements taken at 245 nm. The regression equation for the calibration graph was determined as [*A* = 0.082 *C* / μg mL^-1^ + 0.070 (*r* = 0.998, *n* = 6)], where *A* represents the absorbance value of AVN, and *C* denotes the AVN concentration. Following three replicate measurements, the average AVN content was computed as 101.5 mg per tablet, with an RSD of 1.4 %. Evaluation of the findings suggests similar results were obtained for AVN content using both methods. Furthermore, the results from both the suggested and comparison approaches were subjected to a Student's *t*-test for validation purposes. There were no significant discrepancies between the values acquired using the two approaches and the genuine, labelled values, since the computed (experimental) *t* value = 2.32, at a 95 % confidence range, was lower than the tabulated (theoretical) one (2.78, for *α* = 0.05).

**Figure 8.**
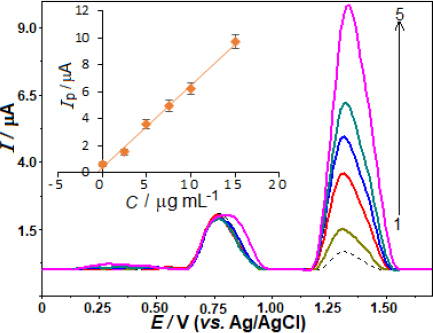
SW voltammograms of the urine sample: the dashed line depicts the diluted urine sample in the presence of 0.5 μg mL^-1^ AVN, with (1-5) showing the standard additions of 2.5, 5.0, 7.5, 10.0 and 15.0 μg mL^−1^ AVN in BR buffer at pH 4.0. Inset shows the result of analysis by standard addition method. Figure 4 shows the other operational parameters.

To evaluate the effectiveness of the proposed approach, the analysis was extended to determine AVN in urine samples. Figure 8 shows SW voltammograms for urine sample analysis, including spiked concentrations of 0.5 μg mL^-1^ in the electrochemical cell. In the unspiked urine sample, an anodic peak appeared around +0.79 V, likely due to UA oxidation. Using the standard addition method, the intensity of the peak increased proportionally with AVN concentration, resulting in a linear calibration plot: *i*_p_ / μA = 0.681 *C* / μg mL^-1^ + 0.315 (*r* = 0.997), as shown in the inset of Figure 8. The electrochemical analysis using this approach for AVN determination showed satisfactory results in terms of recovery percentage and RSD in urine samples. Table 3 highlights robust recovery and RSD values, confirming the potential application of this developed method for the direct quantification of AVN in urine samples.

**Table 3.** Using the developed approach, measurement results for the determination and recovery of AVN from the urine sample.

AVD content, μg mL^-1^		Recovery ± RSD, %
Added	Found^a^	
0.50	0.46	92.0 ±4.3

^a^Calculated by the use of standard addition method.

## Conclusions

The current investigation used an unmodified BDD electrode, which presents a significant departure from the previous analytical procedures in the determination of AVN. This novel approach underscores a departure from the conventional reliance on electrode modification, presenting a fresh perspective in AVN analysis. Importantly, our proposed methodology breaks new ground and showcases several distinct advantages over previous research endeavours. Our approach's inherent practicality, cost-effectiveness, and efficiency stand out prominently, offering a streamlined and resource-efficient avenue for AVN measurement. By eliminating the need for electrode modification, our method simplifies the analytical process, reducing both time and resource requirements while enhancing overall feasibility. The proposed method's applicability for determining AVN in pharmaceutical formulations was successfully demonstrated.
